# CHARTS: a web application for characterizing and comparing tumor subpopulations in publicly available single-cell RNA-seq data sets

**DOI:** 10.1186/s12859-021-04021-x

**Published:** 2021-02-23

**Authors:** Matthew N. Bernstein, Zijian Ni, Michael Collins, Mark E. Burkard, Christina Kendziorski, Ron Stewart

**Affiliations:** 1 Morgridge Institute for Research, Madison, WI 53715 USA; 2 Department of Statistics, University of Wisconsin - Madison, Madison, WI 53706 USA; 3 Department of Medicine, Hematology/Oncology, University of Wisconsin - Madison, Madison, WI 53705 USA; 4 University of Wisconsin Carbone Cancer Center, Madison, WI 53705 USA; 5 Department of Biostatistics and Medical Informatics, University of Wisconsin - Madison, Madison, WI 53792 USA

## Abstract

**Background:**

Single-cell RNA-seq (scRNA-seq) enables the profiling of genome-wide gene expression at the single-cell level and in so doing facilitates insight into and information about cellular heterogeneity within a tissue. This is especially important in cancer, where tumor and tumor microenvironment heterogeneity directly impact development, maintenance, and progression of disease. While publicly available scRNA-seq cancer data sets offer unprecedented opportunity to better understand the mechanisms underlying tumor progression, metastasis, drug resistance, and immune evasion, much of the available information has been underutilized, in part, due to the lack of tools available for aggregating and analysing these data.

**Results:**

We present CHARacterizing Tumor Subpopulations (CHARTS), a web application for exploring publicly available scRNA-seq cancer data sets in the NCBI’s Gene Expression Omnibus. More specifically, CHARTS enables the exploration of individual gene expression, cell type, malignancy-status, differentially expressed genes, and gene set enrichment results in subpopulations of cells across tumors and data sets. Along with the web application, we also make available the backend computational pipeline that was used to produce the analyses that are available for exploration in the web application.

**Conclusion:**

CHARTS is an easy to use, comprehensive platform for exploring single-cell subpopulations within tumors across the ever-growing collection of public scRNA-seq cancer data sets. CHARTS is freely available at charts.morgridge.org.

## Background

Over the past three decades, the cancer research community has amassed large quantities of gene expression data from tumors. The premier example of such data was generated by The Cancer Genome Atlas [4], which generated bulk RNA-seq and microarray data from thousands of tumors across dozens of cancer types. These data have enabled a greater understanding into the molecular biology of cancer and have revealed great heterogeneity not only between cancer types, but also between tumors of the same cancer type [2]. Unfortunately, investigations utilizing this resource are limited by the fact that gene expression was profiled using bulk methods, which measure gene expression on average across thousands, or tens of thousands, of cells in a sample. With the advent of single-cell RNA-seq (scRNA-seq), investigators are now able to measure gene expression at the single-cell level thereby gaining access to the substantial heterogeneity of cells within a tumor and the tumor microenvironment [9]. Publicly available scRNA-seq cancer data sets offer unprecedented opportunity to better understand the mechanisms of tumor progression, metastasis, drug resistance, and immune evasion. However, analyzing these data in the aggregate is challenging, especially for those without strong computational skills. To this end, easy-to-use web-based tools are important for enabling the broader research community to perform integrative analyses and, in doing so, to increase their ability to leverage their knowledge and comprehensively examine scientific and/or clinically relevant hypotheses in multiple data sets.

While a few web-based tools for analyzing scRNA-seq data are available, they are not designed specifically for cancer research or do not easily enable exploration of existing public data sets. For example, recent tools such as Alona [7] and Granatum [29] enable scRNA-seq analysis in the web browser; however, these tools are not cancer-specific and therefore do not enable important cancer-specific tasks such as classifying cells as being either transformed malignant cells or untransformed cells of the tumor microenvironment. Furthermore, these tools do not enable exploration of preprocessed, publicly available scRNA-seq data sets. Another tool, GREIN [16], enables exploration of public gene expression data, but it is neither single-cell specific nor cancer-specific and, consequently, does not implement features necessary for single-cell analysis such as cell type identification, clustering, or gene set enrichment, nor does it implement cancer-specific analyses such as malignancy classification. CancerSEA [27] enables exploration of gene set enrichment scores for gene sets pertaining to cancer-related processes, but does not enable visualization, differential expression, or cell type identification. In short, while web-based tools exist for exploring expression data, most do not allow for detailed analysis of scRNA-seq data across diverse tumors and data sets.

To address this gap, we present CHARacterizing Tumor Subpopulations (CHARTS), a web application and associated computational pipeline for analyzing and characterizing publicly available cancer scRNA-seq data sets. As described in detail below, for each tumor in its database, CHARTS identifies clusters and enables exploration via interactive dimension-reduction methods. Derived clusters are annotated with cell types from the Cell Ontology [1] via CellO [3], with information provided on the probability of the specific cell type as well as its ancestors. For example, the data may provide substantial evidence to classify cells within a cluster as T cells, but less evidence may be available to classify cells into more specific functional groups (e.g. helper or memory T cells). In addition, for each cluster within each tumor, enrichment of genes involved in biological processes and pathways is provided. Genes that are differentially expressed between the cluster and others are also available. Finally, CHARTS can be used to distinguish malignant vs. non-malignant cells allowing for precise exploration into the interactions between cell subpopulations within the tumor microenvironment. CHARTS currently enables exploration of 198 tumors across 15 cancer types, and data is being continually added. CHARTS is freely available at charts.morgridge.org.

## Implementation

### Data set selection and preprocessing

We used the curated database describing single-cell RNA-seq data sets by Svensson et al. [22] to identify single-cell cancer data sets that are publicly available in the Gene Expression Omnibus (GEO) [6]. We selected all studies that sequenced primary tumor samples (*i.e.*, non-cell line and non-xenograft samples) that consist of cells from the full tumor microenvironment (*i.e.*, that do not select for a specific cell type). For each study, we wrote a custom script for separating cells by their tumor and normalizing the data by estimating the transcripts per million (TPM) for each gene and then computing log(TPM + 1). We note that for droplet-based assays that sequence transcripts only from their end, such as Chromium 10x, an estimate of each gene’s TPM in a cell can be obtained by dividing each gene’s UMI count by the total UMI count in the cell and multiplying by one million. By estimating TPM, we measure gene expression using consistent units across assays. We exclude samples for which their corresponding GEO entry does not include enough information to estimate TPMs. Specifically, we exclude data sets originating from full length assays (*i.e.,* where reads can originate from anywhere along the full length of the transcript), such as Smart-seq2 [21], if their GEO entry either does not include estimates of TPM, or includes counts, but does not include an estimate of each gene’s expected length, which is required for estimating TPM. This process selected 198 tumors across 18 studies comprising a total of 259,488 cells.

**Fig. 1:**
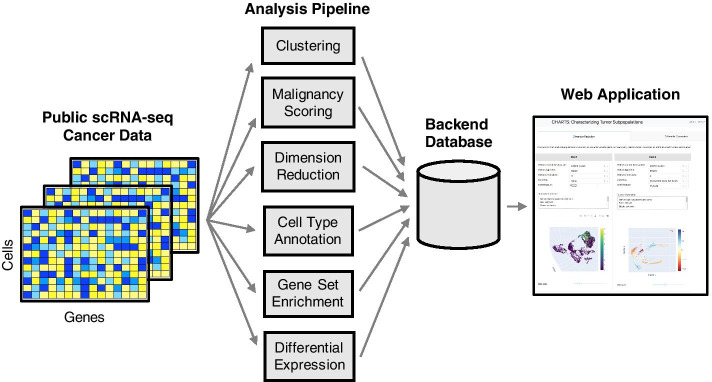
Overview. A schematic diagram of the CHARTS pipeline. Public scRNA-seq data sets are collected and analyzed with a custom pipeline. This pipeline computes clusters, malignancy scores, dimension reduction transformations, cell type annotations, gene set enrichment scores, and differentially expressed genes for each cluster. Results are stored in a backend database and are accessed from the frontend web application

These datasets were then processed with an offline computational pipeline. This pipeline implements a number of analyses in order to enable comprehensive characterization and comparison of tumor subpopulations within and between tumors (Fig. 1). All analysis output is stored in a backend database, which is quickly and easily accessible to a user through a frontend web application.

### Dimension reduction

A user may construct interactive dimension-reduction scatterplots using two or three-dimensional UMAP [17] or PHATE [18] results. Each cell can be colored by the expression of a user-specified gene, cluster, malignancy score, cell type, or gene set enrichment. Two scatterplots are placed side-by-side enabling users to compare two characteristics (e.g. two different genes’ expression values or a gene’s expression value and the predicted cell types) within the same tumor or to compare a single characteristic (e.g. a single gene’s expression values) between two tumors. For computational efficiency, we perform UMAP on the first 50 principal components after running principal components analysis (PCA).

### Clustering

Clustering is performed using the Leiden community detection algorithm [25], as implemented in Python’s Scanpy library [26]. Determining the optimal clustering is a challenging open problem in single-cell RNA-seq analysis [11]. If clustering is too coarse, multiple cell types may be erroneously combined into a single cluster. Similarly, if clustering is too fine, homogenous populations of cells may be broken into multiple clusters. Rather than choose a single clustering granularity for all tumors, CHARTS provides clusterings at three levels of granularity via three values for Leiden’s resolution parameter (0.5, 1.0, and 2.0). Clustering is performed on the first 50 principal components after running PCA. The web interface enables a user to select a level of clustering granularity for exploring each tumor.

### Cell type annotation

For each clustering granularity, each cluster is annotated with cell types from the Cell Ontology [1] via CellO [3]. The Cell Ontology is a hierarchically structured knowledgebase of known cell types. Specifically, the Cell Ontology forms a directed acyclic graph (DAG) where edges in the graph represent “is a” relationships. Because of this DAG structure, each cell is assigned to a specific cell type as well as all ancestors of this specific cell type within the DAG. CellO was executed using the isotonic regression correction algorithm. CHARTS exposes both CellO’s binary cell type decisions for each cell type as well as CellO’s estimated probability that each cell is of a given type.

### Gene set enrichment

Each cluster’s mean gene expression profile is scored for enrichment of gene sets describing molecular processes. Specifically, CHARTS uses GSVA [10] to score each cluster for enrichment of gene sets in the hallmark gene set collection from MSigDB [14] and the gene set collection used by CancerSEA. Specifically, we treat the TPM expression measurements as being distributed according to a log-normal distribution [8] and use the Gaussian kernel in the GSVA algorithm.

### Malignancy status

Each cell is assigned a malignancy score that describes the likelihood that the cell is malignant. The malignancy scoring approach builds upon the approaches used by Tirosh et al. [24] and Couturier et al. [5] for classifying cells as either transformed, malignant cells or untransformed cells within the tumor microenvironment (Additional file 1: Supplementary Methods).

### Differential expression and cluster comparison

For each cluster within each tumor, CHARTS uses a Wilcoxon rank-sum test, as implemented in Scanpy, to compute the set of genes differentially expressed in the given cluster versus cells outside the cluster within the given tumor. CHARTS presents the top 50 genes ranked according to their significance. In addition to differential expression analysis, CHARTS also presents boxplots and violin plots for comparing the distribution of gene expression for a user-selected gene between clusters.

## Results

Two case studies demonstrate how CHARTS can be used, both to examine and generate new hypotheses.

### Case study: dysfunctional CD8 + T cells in lung adenocarcinoma

**Fig. 2:**
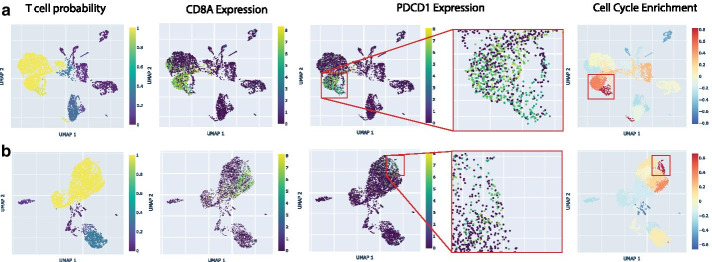
Dysfunctional CD8 + T Cells in Lung Adenocarcinoma. For lung adenocarcinoma tumors LX682 (**a**) and LX676 (**b**), we used CHARTS to visualize the probability that each cell is a T cell as well as expression of CD8A, expression of the dysfunctional CD8 + T cell marker PDCD1, and each cell’s enrichment score for genes in CancerSEA’s cell cycle gene set as produced by GSVA

Investigators have recently reported a dysfunctional population of CD8 + T cells in lung cancer [23] and melanoma [15] that express genes associated with immune suppression. In some melanoma samples, this population was also found to be highly proliferative [15]. We used CHARTS to explore whether this dysfunctional state was common across the majority of CD8 + T cells, and to evaluate whether dysfunctional CD8 + T cells were also highly proliferative. We used both CellO’s classification results and CD8 expression to find the CD8 + T cell population. We found that in the majority of lung adenocarcinomas, only a subset of CD8 + T cells express marker genes for this dysfunctional state. Two adenocarcinomas from [13] are shown in Fig. 2. Using the gene set enrichment feature of CHARTS, we further found that dysfunctional cells are enriched for cell cycle genes, which may indicate that these dysfunctional CD8 + T cells are highly proliferative in lung adenocarcinoma, as has been recently observed in melanoma.

### Case study: monocarboxylate transporters in glioblastoma

**Fig. 3:**
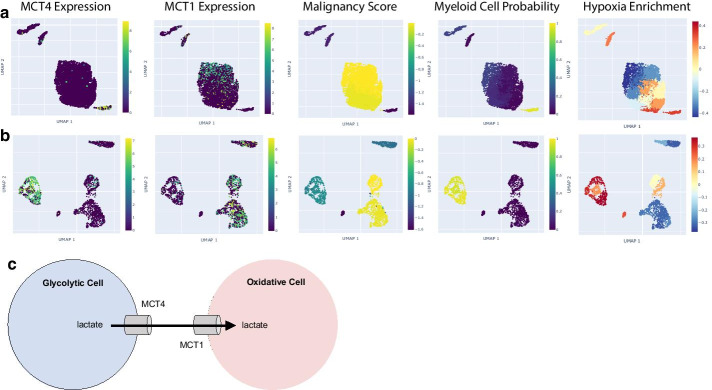
Monocarboxylate Transporter Expression in Glioblastoma. For glioblastoma tumors PJ025 from Yuan et al. [28] (**a**) and MGH125 from Neftel et al. [19] (**b**), we used CHARTS to visualize the expression of MCT4 (gene symbol “SLC16A3” in the CHARTS application), the expression of MCT1 (gene symbol “SLC16A1” in the CHARTS application), malignancy score, the probability that each cell is a myeloid cell, and each cell’s enrichment score for genes in the Hallmark hypoxia gene set as produced by GSVA. (**C**) A schematic illustration of the metabolic symbiosis between hypoxic, glycolytic tumor cells expressing MCT4 and oxidative tumor cells expressing MCT1

We investigated the expression of MCT4, a prognostic biomarker of glioblastoma aggression [12, 30]. Using CHARTS, we found that MCT4 tended to be expressed in the myeloid tumor-infiltrating immune cells. Two tumors, from Yuan et al. [28] and Neftel et al. [19], are shown in Fig. 3a and b respectively. While MCT4 is known to be involved in a metabolic symbiosis between hypoxic tumor cells, which express MCT4 to expel lactate, and oxidative tumor cells, which express MCT1 to intake lactate [20] (Fig. 3c), the specific cell types expressing MCT4 in glioblastoma have not been well characterized. We used CHARTS to determine which cells express MCT1 in glioblastoma and found that this gene was primarily expressed in cells with high malignancy scores (Fig. 3a, b). Using the gene set enrichment feature of CHARTS, we observed that cells expressing MCT1 tended to express genes enriched for hypoxia, whereas cells expressing MCT4 tended to express genes that were less enriched for hypoxia (Fig. 3a, b). This observation indicates a possible metabolic symbiosis between malignant cells and myeloid cells in the tumor microenvironment of glioblastoma, which to the best of our knowledge, has not been well characterized.

## Conclusion

In this work, we present CHARTS: a comprehensive framework for exploring single-cell subpopulations within tumors and the tumor microenvironment across ever-growing data sets. CHARTS can be used to develop and explore new hypotheses underlying tumor progression, drug resistance, and immune evasion. Specifically, CHARTS exposes the results of an analysis pipeline executed on publicly available data from GEO.

There are a number of avenues that would benefit from further investigation. First, we note that CHARTS presents the results of a static analysis pipeline that was executed on diverse single cell datasets. Thus, individual datasets may benefit from tweaking the pipeline’s parameters on an individual basis (e.g., parameters for clustering or dimensionality reduction). Future work will entail devising methods for fine-tuning the parameters used to process each dataset.

We note that this analysis pipeline was executed on published gene expression matrices and did not involve processing the raw sequencing reads. Thus, there may be variability between data sets due to the varying methods employed by the data submitters for alignment, gene expression quantification, and quality control. Future work will seek to remove some of this variability by starting with the raw reads from each dataset, rather than the expression matrices, and uniformly quantifying expression and filtering cells across datasets.

### Availability and requirements

- Project name: CHARTS
- Project home page: https://charts.morgridge.org
- Operating systems(s): The web application is platform independent. The backend pipeline has been tested only on Linux and MacOS.
- Programming language: Python
- Other requirements: The backend pipeline requires Python 3.6 or higher.
- License: MIT
- Any restrictions to use by non-academics: None

## Supplementary Information

**Additional file 1**. Supplemental Methods. Description and benchmarking of malignancy score.

## Data Availability

Code implementing the web application and offline data analysis pipeline is available at https://github.com/stewart-lab/CHARTS.

## Abbreviations

scRNA-seq: Single-cell RNA sequencing
GEO: Gene Expression Omnibus
DAG: Directed acyclic graph
TPM: Transcripts per million
